# Integrated Transcriptomic and Metabolomic Analyses Reveal the Whitening of Perirenal Brown Adipose Tissue in Yak Calves

**DOI:** 10.3390/ani16203219

**Published:** 2026-10-13

**Authors:** Fuju Chen, Yu Hu, Lizhuang Hao, Zhenshuo Bai

**Affiliations:** 1 College of Agriculture and Animal Husbandry, Qinghai University, Xining 810016, China; 2 Key Laboratory of Plateau Grazing Animal Nutrition and Feed Science of Qinghai Province, Qinghai University, Xining 810016, China

**Keywords:** yak calves, perirenal adipose tissue, BAT whitening, thermogenesis, lipid metabolism

## Abstract

Brown adipose tissue (BAT) is essential for neonatal thermogenesis; however, in yak, the adipose tissue exhibits age-related changes from a brown-like to a white-like phenotype between postnatal day 1 (P1) and day 30 (P30). This exploratory study aimed to characterize the molecular and metabolic changes underlying this postnatal transition, with a particular focus on thermogenesis and lipid metabolism. Perirenal adipose tissue from yak calves at P1 and P30 was analyzed using phenotypic, transcriptomic (*n* = 3 per group), and metabolomic (*n* = 6 per group) approaches. Our results showed that multilocular adipocytes were abundant at P1, whereas unilocular adipocytes were predominant by P30, accompanied by significantly increased triglyceride content (*p* < 0.05). Transcriptomic and metabolomic analyses identified a total of 1000 differentially expressed genes (DEGs) and 17 differential metabolites between the two stages. From the DEGs, 13 thermogenesis-related genes and 24 lipid metabolism-related genes were selected based on functional relevance, and all 17 differential metabolites were analyzed. Integrative analysis of the transcriptomic and metabolomic datasets further identified 14 key genes related to thermogenesis and lipid metabolism and 6 differential metabolites potentially associated with these metabolic pathways. Among them, *CDS*2, *GPAM*, *AGPAT*2, *CPT*1*C*, *GADD*45*B*, *PLIN*1, and metabolites phosphatidylethanolamine (PE), phosphatidic acid (PA), and oxidized phosphatidylserine (PS) may serve as a potential link between lipid deposition and thermogenic metabolism. Fatty acid profiling showed that n-3 polyunsaturated fatty acids (n-3 PUFAs), such as eicosapentaenoic acid (EPA, 20:5 n-3) and docosahexaenoic acid (DHA, 22:6 n-3) were enriched in BAT, while stearic acid (SA, 18:0) and oleic acid (OA, 18:1 n-9) were enriched in WAT. These findings suggest age-related changes in the molecular and metabolic characteristics of postnatal BAT from a thermogenic phenotype toward a lipid-storage state, consistent with a functional transition during adipose tissue development. This provides a molecular framework for understanding BAT phenotypic changes in neonatal yaks and identifies candidate targets for future studies investigating the regulation of thermogenesis and lipid metabolism in plateau ruminants.

## Simple Summary

Brown adipose tissue plays an important role in maintaining body temperature in newborn yak calves exposed to the cold environment of the Qinghai–Tibet Plateau. In this study, we combined transcriptomic and metabolomic analyses to investigate the postnatal whitening of perirenal brown adipose tissue in yak calves. We found that adipose tissue changed from a brown-like phenotype on day 1 (P1) to a white-like phenotype by day 30 (P30), accompanied by changes in genes and metabolites related to thermogenesis, mitochondrial energy metabolism, and lipid synthesis and storage. We also identified distinct fatty acid profiles between the two developmental stages. These findings provide new insights into the molecular and metabolic changes associated with brown adipose tissue whitening in yak calves and contribute to a better understanding of adipose tissue development and cold adaptation in plateau yaks.

## 1. Introduction

The Qinghai–Tibet Plateau, the highest and one of the most extreme regions in the world, has an average altitude exceeding 4000 m and an annual mean temperature below 0 °C. Characterized by harsh conditions including hypoxia and cold, the plateau poses significant challenges to the survival and reproduction of native animals. The yak (*Bos grunniens*), an indigenous ruminant uniquely adapted to this region, has evolved multidimensional cold adaptation mechanisms through long-term natural selection, making it an ideal model for studying extreme cold environmental adaptation in plateau mammals [1]. Yak calves are a vital resource for livestock production in alpine pastoral areas, and their survival rate directly affects herders’ economic income and the sustainability of the industry. Newborn yak calves face significant thermoregulatory challenges during the transition from the uterus to the external environment, where insufficient heat production can lead to hypothermia, growth retardation, and increased susceptibility to diseases [2].

Adipose tissue plays a dual role as an energy reservoir and an endocrine organ, regulating lipid metabolism, inflammation, and glucose homeostasis [3,4]. Mammalian adipose tissue is broadly classified into two main types: white adipose tissue (WAT) and brown adipose tissue (BAT). White adipocytes contain a unilocular lipid droplet and function primarily in lipid storage and mobilization. In contrast, brown adipocytes possess high mitochondrial content and mediate non-shivering thermogenesis (NST) primarily through uncoupling protein 1 (UCP1), a mitochondrial protein that uncouples respiration from ATP synthesis to generate heat. Therefore, BAT plays a pivotal role in maintaining core body temperature, particularly in neonates [5,6,7]. Recent studies have further identified beige adipocytes, which appear within WAT in response to cold exposure or β-adrenergic receptor activation, a phenomenon known as the browning of WAT [7,8].

In many mammals, including humans [9], goats [10,11,12], sheep [13], rabbits [14,15], and calves [16,17,18], BAT is particularly abundant during early life and plays an important role in coping with cold exposure. Consequently, neonatal survival is closely related to the NST capacity of BAT. After birth, skeletal muscle also contributes to heat production through shivering thermogenesis, while BAT gradually declines and acquire white-like adipocyte characteristics during postnatal development. However, the timing and extent of BAT whitening vary among species. For example, in humans, brown adipocytes are mainly found in interscapular BAT depots and are largely restricted to infancy [9]. In Dazu black goat, the perirenal adipose tissue changes from reddish-brown in newborns at day 0 (D0) to milky white at day 28 (D28), and the adipocytes gradually transition from multilocular small lipid droplets at D0 to unilocular large lipid droplets at D28 [11]. In sheep, perirenal fat during the first month after birth undergoes two distinct developmental stages: the first stage occurs at 4–7 days of age, when its main function is heat production via increased UCP activity; the second stage occurs at 7–30 days of age and is characterized by decreased UCP mRNA content and increased lipid deposition [13]. In bovines, perirenal BAT develops rapidly at 194–240 days of gestation and is then replaced by WAT approximately 2–30 days after birth [13]. This conversion is marked by diminished thermogenic capacity, enlarged lipid droplets, and reduced UCP1 expression [19]. However, the regulatory mechanisms underlying this BAT whitening transition remain poorly understood.

Several recent studies have attempted to address this gap. For instance, aging has been shown to affect the rhythmicity of various lipid classes, including glycerolipids, glycerophospholipids, sterol lipids, and sphingolipids in both BAT and WAT of mice [20]. A previous study on goat perirenal adipose tissue reported that marker genes such as *UCP*1, *CIDEA*, and *COX*7*A*1 were highly expressed in BAT on day 0, whereas *NRIP*1, *LPL*, *LEP*, *EBF*3, *SLC*7*A*10, and *RBL*1 were markedly upregulated in WAT on day 28, indicating the existence of distinct transcriptional signatures between the two adipose phenotypes [11]. Wang et al. [21] further identified changes in fatty acid desaturation and degradation pathways during the BAT-to-WAT transition in goats using integrated transcriptomic and metabolomic analyses. Zhao et al. [11] performed global gene expression profiling of perirenal brown adipose tissue whitening in goats and raised the question of whether this BAT-to-WAT change is due to transdifferentiation of mature adipocytes or to adipocyte heterogeneity, in which low- and high-thermogenic brown adipocyte subpopulations coexist.

In yak calves, we previously examined the postnatal development of interscapular and subcutaneous adipose tissue at 1, 7, and 30 days of age using light microscopy, transmission electron microscopy, real-timeqRT-PCR, Western blotting, and immunohistochemistry [19]. We found typical multilocular adipocytes in the interscapular and subcutaneous adipose tissue of yak calves. Moreover, many multilocular adipocytes were transformed into unilocular adipocytes at 30 days of age, but some multilocular adipocytes remained. However, the thermogenic function of these multilocular adipocytes, as well as the molecular mechanisms that regulate the cellular transition from multilocular to unilocular adipocytes after birth, remains unclear. Furthermore, because goats living in southern regions with milder winters and yak calves living in extremely cold northern regions inhabit different environments, it remains unclear whether the postnatal development of BAT and its underlying mechanisms are the same in these animals. Elucidating BAT thermogenesis, its developmental changes, and the underlying mechanisms in animals from different living environments is of great significance for understanding BAT function.

Therefore, the objective of this study was to characterize the molecular and metabolic changes underlying the postnatal transition of perirenal brown adipose tissue to white adipose tissue in yak calves, with a particular focus on changes related to thermogenesis and lipid metabolism.

## 2. Materials and Methods

### 2.1. Animal Ethics Statement

All animal experiments were reviewed and approved by the Institutional Animal Care and Use Committee of Qinghai University (Protocol Code: SL 2023041).

### 2.2. Experimental Animals and Sample Collection

The yak calves used in this study were obtained from the Datong Yak Breeding Centre in Qinghai Province, China (3100 m above sea level; coordinates 101.400179° E, 37.252646° N), as previously described [22]. The mother yaks grazed naturally on alpine pasture without supplemental feeding, and the yak calves followed their mothers after birth. Calves were naturally suckled by their dams, including colostrum intake during the first hours after birth; no additional milk replacer or feeding was provided. No additional housing was provided. All calves were clinically healthy at birth and throughout the study period, as assessed by daily clinical observation. In April 2023, twelve healthy male yak calves were selected, comprising six individuals at postnatal day 1 (P1, *n* = 6, mean body weight, 14.32 kg) and six at postnatal day 30 (P30, *n* = 6, mean body weight, 26.74 kg). The mean ambient temperature during sample collection was −2 °C.

All animals were euthanized by jugular injection of an overdose of sodium pentobarbital (80 mg/kg body weight; Beuthanasia-D Special; Schering-Plough Animal Health). Immediately following euthanasia, perirenal adipose tissue was collected. A portion of each sample was fixed in cold 4% paraformaldehyde in 0.1 M phosphate-buffered saline (pH 7.4) for histological analysis. The remaining tissue was frozen in liquid nitrogen and stored at −80 °C for subsequent metabolomics, fatty acid analysis, and transcriptomic sequencing.

Because the amount of perirenal adipose tissue was relatively limited, especially at P1, tissue from all six calves in each group was first used for untargeted metabolomics and fatty acid profiling (*n* = 6 per group). From the remaining tissue, samples from three calves per group were used for RNA-seq, and the same three calves were used for RT-qPCR validation of key transcriptomic findings. Four calves per group were used for histological evaluation and triglyceride content determination. One calf per group was used for both transcriptomic and histological analyses, so the total number of animals remained six per group. The different sample sizes across assays were therefore due to limited tissue availability and the amount of tissue required for each analysis.

### 2.3. Histological Assessment via Hematoxylin–Eosin (H&E) Staining

Perirenal adipose tissue from four yak calves per age group (*n* = 4/group) were processed for hematoxylin–eosin (H&E) staining. Tissues were dehydrated through a graded ethanol series, embedded in paraffin, and sectioned at a thickness of 6 μm. H&E staining was performed according to a previously described method [19,22]. Adipocytes were classified according to previously established criteria [19]: brown (multilocular) adipocytes were defined as cells containing multiple small lipid droplets with intense eosin staining, whereas white (unilocular) adipocytes were defined as cells containing a single large lipid droplet with weaker eosin staining. Observations were made using an upright optical microscope (Eclipse E100, Nikon, Tokyo, Japan). The densities of multilocular and unilocular adipocytes (cells/μm^2^) were determined by counting cells in five randomly selected fields per section, with five sections per animal, using ImageJ software v1.54f (National Institutes of Health, Bethesda, MD, USA) [19].

### 2.4. Triglyceride Content Determination

Triglyceride (TG) content in perirenal adipose tissue was measured using a commercial assay kit (Nanjing Jiancheng Bioengineering Institute, Nanjing, China) following the manufacturer’s instructions [23]. Measurements were performed on samples from four animals per age group (*n* = 4/group). The same four animals used for histological analysis were used for TG determination to maintain consistency between the morphological and biochemical assessments.

### 2.5. Transcriptomic Analysis

#### 2.5.1. RNA Extraction and Library Construction

To characterize transcriptional changes accompanying the age-related changes from a brown-like to a white-like phenotype, perirenal adipose tissue samples were collected from three yak calves at P1 (P1_1, P1_2, and P1_3) and three at P30 (P30_1, P30_2, and P30_3), with each calf representing one independent biological replicate. RNA sequencing services were provided by Novogene Co., Ltd. (Beijing, China). Total RNA was extracted using TRIzol reagent (Tiangen, Beijing, China) according to the manufacturer’s protocol. RNA purity (OD260/280) and concentration were assessed using a NanoDrop 2000 spectrophotometer (Thermo Fisher Scientific, Waltham, MA, USA). Only samples with an OD260/280 ratio between 1.8 and 2.0 and an RNA integrity number (RIN) > 7 were considered acceptable for downstream applications. Qualified RNA samples were used for mRNA isolation and cDNA synthesis. Sequencing libraries were subsequently constructed through end repair, A-tailing, adapter ligation, and PCR amplification. After library quality control, the qualified libraries were subjected to cluster generation and paired-end sequencing on an Illumina NovaSeq 6000 platform (Illumina, San Diego, CA, USA).

#### 2.5.2. Sequencing Data Processing and Annotation

Raw sequencing data were filtered to remove low-quality sequences, and the resulting clean reads were assessed for error rates and GC content distributions. Clean reads were aligned to the reference genome using HISAT2 (v2.0.5), and transcript assembly was performed with StringTie. Novel transcripts or genes were identified by comparing assembled transcripts against genome annotation using GFF. Gene expression levels were quantified as read counts using HTSeq (v2.0.5) and subsequently normalized to fragments per kilobase of transcript per million mapped reads (FPKM). Principal component analysis (PCA) was performed using R (v4.4.1) to evaluate biological reproducibility among samples.

#### 2.5.3. Differential Expression Analysis and Functional Enrichment

Differentially expressed genes (DEGs) between P1 and P30 groups were identified using DESeq2 (v1.30.0) in R [24], with thresholds set at |log2 (fold change)| ≥ 1 and Benjamini–Hochberg (BH)-adjusted *p*-value (FDR) < 0.05. Clustering of gene expression patterns was visualized using the pheatmap package (v1.0.8). Gene Ontology (GO) and Kyoto Encyclopedia of genes and genomes (KEGG) pathway enrichment analyses were implemented using clusterProfiler (3.8.1) [25]. For enrichment analysis, the background gene set consisted of all genes that were detected and included in the differential expression analysis. For enrichment analysis, the background gene set consisted of all genes that were detected and included in the differential expression analysis. Multiple-testing correction was performed using the Benjamini–Hochberg (BH) method, and adjusted *p*-values < 0.05 were considered statistically significant for enriched terms.

To validate the transcriptomic data, real-time quantitative PCR (RT-qPCR) was performed on five selected genes. Briefly, total RNA (1 μg) was reverse-transcribed using the PrimeScript™ RT Reagent Kit with gDNA Eraser (Takara, Tokyo, Japan) according to the manufacturer’s instructions. RT-qPCR reactions were performed using PowerUP™ SYBR^®^ Green Master Mix (Applied Biosystems, Waltham, MA, USA) on samples from three animals per age group, with each sample analyzed in triplicate. The thermal cycling conditions were as follows: 95 °C for 30 s, followed by 40 cycles of 95 °C for 5 s and 55–58 °C for 30 s, with a final extension at 72 °C for 30 s. Relative gene expression levels were calculated using the 2^−ΔΔCt^ method, with β-actin as the reference gene. The stability of β-actin expression across age groups was assessed by comparing its Ct values between the P1 and P30 groups, and no significant intergroup difference was observed. Amplification efficiencies of all target and reference gene primer pairs were determined from standard curves before sample analysis and ranged from 90% to 110%, which was within the generally accepted range for RT-qPCR assays. Gene primer sequences were synthesized by Tsingke Biotech Co., Ltd. (Beijing, China) (Supplementary Table S1), and their amplification efficiency was pre-validated prior to use.

### 2.6. Untargeted Metabolomics Sequencing and Analysis

Untargeted metabolomics was performed on perirenal adipose tissue samples from six animals per age group (P1_1-P1_6 and P30_1-P30_6; Total *n* = 12). Untargeted metabolomics analysis was provided by Oebiotech Co., Ltd. (Shanghai, China). Sample preparation and ultra-performance liquid chromatography-tandem mass spectrometry (UPLC-MS/MS) analysis were conducted according to a previously described protocol [23]. Briefly, 30 mg of perirenal adipose tissue sample was weighed into a 1.5 mL EP tube, two small steel beads were added, followed by 400 μL methanol-water solution (V:V = 4:1, containing mixed internal standards at 4 μg/mL). The tube was pre-cooled at −40 °C for 2 min, and then homogenized in a tissue grinder at 60 Hz for 2 min. Samples were further extracted by sonication in an ice-water bath for 10 min and incubated at −40 °C for 2 h. After centrifugation at 13,000 rpm for 20 min at 4 °C, 150 μL supernatant was collected with a syringe, filtered through a 0.22 μm organic phase syringe filter, transferred into an LC vial, and stored at −80 °C prior to LC-MS analysis. All extraction reagents were pre-cooled at −20 °C before use. Pooled quality control (QC) samples were prepared by mixing equal volumes of extracts from all experimental samples. Chromatographic separation was performed on a Waters ACQUITY UPLC I-Class plus system (Waters, Milford, MA, USA) coupled with a Thermo Q Exactive Plus high-resolution mass spectrometer (Thermo Fisher Scientific, Waltham, MA, USA). Chromatographic separation was performed using a Waters ACQUITY UPLC HSS T3 C18 column (1.8 μm, 2.1 mm × 100 mm), with 0.1% formic acid in ultrapure water (A) and 0.1% formic acid in acetonitrile (B) as the mobile phases for gradient elution. The flow rate was 0.4 mL/min, the column temperature was 40 °C, and the injection volume was 3 μL. The gradient elution program was as follows: 0–2 min, 5%B; 2–4 min, 5–30% B; 8–10 min, 50–80% B; 10–14 min, 80–100% B; 14–15 min, 100% B; and 15.1–16 min, returned to 5% B.

Mass spectrometry detection was performed using an electrospray ionization (ESI) source in both positive and negative ion modes. The ion source temperature was set at 500 °C, and the spray voltages were 3800 V and 3000 V, respectively. Ion source gas I (GSI), ion source gasII(GSII), and curtain gas (CUR) were set at 50, 50, and 25 psi respectively, and the collision gas (CAD) was set to high. QC samples were injected periodically throughout the analytical sequence. Both PCA and pairwise Pearson correlation analysis were applied to evaluate data quality. The pooled QC samples clustered tightly in the PCA score plot, and pairwise Pearson correlation coefficients among QC replicates were greater than 0.995, indicating high analytical stability and good reproducibility of the metabolomic data. Multivariate data analysis and modeling were conducted using R (v4.0.3) and the ropls package (v1.36.0). PCA and orthogonal partial least squares discriminant analysis (OPLS-DA) were conducted to visualize sample distribution and maximize group separation [26,27], with model performance validated by 200 permutation tests to exclude overfitting. Unpaired Student’s *t*-test was used to calculate raw *p*-values between the two age groups. The Benjamini–Hochberg method was used for multiple-testing correction of *p*-values, yielding FDR-adjusted Q-values. Differential metabolites (DMs) were identified by matching accurate mass, MS/MS fragmentation patterns, and retention time against the KEGG, HMDB, and ChEBI databases. Metabolites meeting the criteria of fold change (FC) > 1.2 or FC < 1/1.2, VIP > 1.0 from the OPLS-DA model, and Q-value < 0.05 were defined as DMs and selected for downstream analyses [28,29]. The selected DMs were subjected to cluster analysis using R, and key DMs were analyzed for KEGG pathway enrichment using the KEGG database to construct relevant metabolic pathways [30].

### 2.7. Integrative Analysis of Transcriptomic and Metabolomic Datasets

Transcriptome and metabolome KEGG enrichment analyses were performed to identify key functional pathways associated with phenotypic differences between groups. Only samples with both transcriptomic and metabolomic datasets (six samples, *n* = 3 per group) were retained for integrated correlation analysis. Based on the integrated analysis, 14 key genes and 6 key differential metabolites were selected according to their functional relevance to thermogenesis and lipid metabolism. Pearson correlation analysis was then performed for all possible gene–metabolite combinations between these 14 genes and 6 metabolites, resulting in a total of 84 gene–metabolite pairs (14 × 6) tested. To control for false-positive rates, the Benjamini–Hochberg method was applied for multiple-testing correction, and FDR-adjusted Q-values were calculated. Gene–metabolite pairs with |Pearson correlation coefficient| > 0.9 and Q-value < 0.05 were considered statistically significant. Correlation clustering heatmaps were generated to visualize the overall association patterns. Furthermore, we utilized Cytoscape software (v3.10.1) to construct a multi-omics gene–metabolite regulatory association network based on the significant correlation pairs. It should be noted that correlation analysis was performed across two groups, and the observed associations may partially reflect inter-group divergence rather than pure within-group biological covariation [31]. Therefore, all correlation-based results in this study are interpreted as exploratory molecular associations rather than definitive regulatory relationships.

### 2.8. Fatty Acid Extraction and Gas Chromatography Analysis

Fatty acid profiling was performed on perirenal adipose tissue samples from six animals per age group (*n* = 6 per group). Total fatty acids released by acid hydrolysis were extracted and analyzed following a previously described protocol [32]. Briefly, 25 μL 2 mM docosanoic-22,22,22-D3 acid internal standard was added to the adipose tissue homogenate containing 50 μg protein in a glass centrifuge tube. After mixing, 1 mL of CH_3_CN/37% HCl (2:1, *v*/*v*) was added to each tube, vortexed at full speed for 1 min, and heated at 90 °C for 1 h. The tubes were then cooled to room temperature, 2 mL of hexane was added, vortexed at full speed for 10 s, and centrifuged at 3000 rpm for 5 min. The lower aqueous-protein layer was discarded, and the upper hexane-rich organic phase was transferred to a new vial, dried under a stream of nitrogen, reconstituted in 1 mL of methanol with 0.25% NH_4_OH, and loop-injected (10 μL) into a Thermo Fisher LTQ Orbitrap Velos for high-resolution electrospray ionization mass spectrometric analysis.

For quantitative analysis, fatty acid methyl esters (FAMEs) were prepared and separated on an Agilent 7820A gas chromatograph equipped with an HP-88 capillary column (100 m × 0.25 mm × 0.20 μm) (Agilent Technologies, Santa Clara, CA, USA). The carrier gas was high-purity nitrogen (purity ≥ 99.99%) at 1.0 mL/min with a split ratio of 50:1. The injector temperature was 250 °C, the detector temperature was 280 °C, and the injection volume was 1 μL. The column temperature program was as follows: held at 80 °C for 2 min, raised to 180 °C at 10 °C/min and held for 1 min, then raised to 220 °C at 5 °C/min and held for 20 min. Qualitative analysis was performed by comparing retention times with fatty acid methyl ester standards (47015-U, Sigma-Aldrich, St. Louis, MO, USA). The relative percentage content of each fatty acid was calculated using the area normalization method.

### 2.9. Statistical Analysis

Statistical analyses were performed using SPSS software (Ver.17.0, SPSS Inc., Chicago, IL, USA). The individual animal was considered the experimental unit, and technical replicates from the same animal were averaged before statistical analysis and were not treated as independent biological replicates. Data from adipocyte density, triglyceride content, fatty acid levels, and RT-qPCR analyses are presented as mean ± standard error of the mean (SEM). Comparisons between groups were conducted using one-way analysis of variance (ANOVA). The normality of the data was assessed using the Shapiro–Wilk test, and homogeneity of variances was assessed using Levene’s test before ANOVA. For comparisons involving multiple individual fatty acids, *p*-values were adjusted for multiple testing using the Benjamini–Hochberg method, and FDR-adjusted *p*-values < 0.05 were considered statistically significant. For other comparisons, a *p*-value < 0.05 was considered statistically significant.

## 3. Results

### 3.1. Morphological Transition and Increased Lipid Accumulation in Perirenal Adipose Tissue

**Figure 1:**
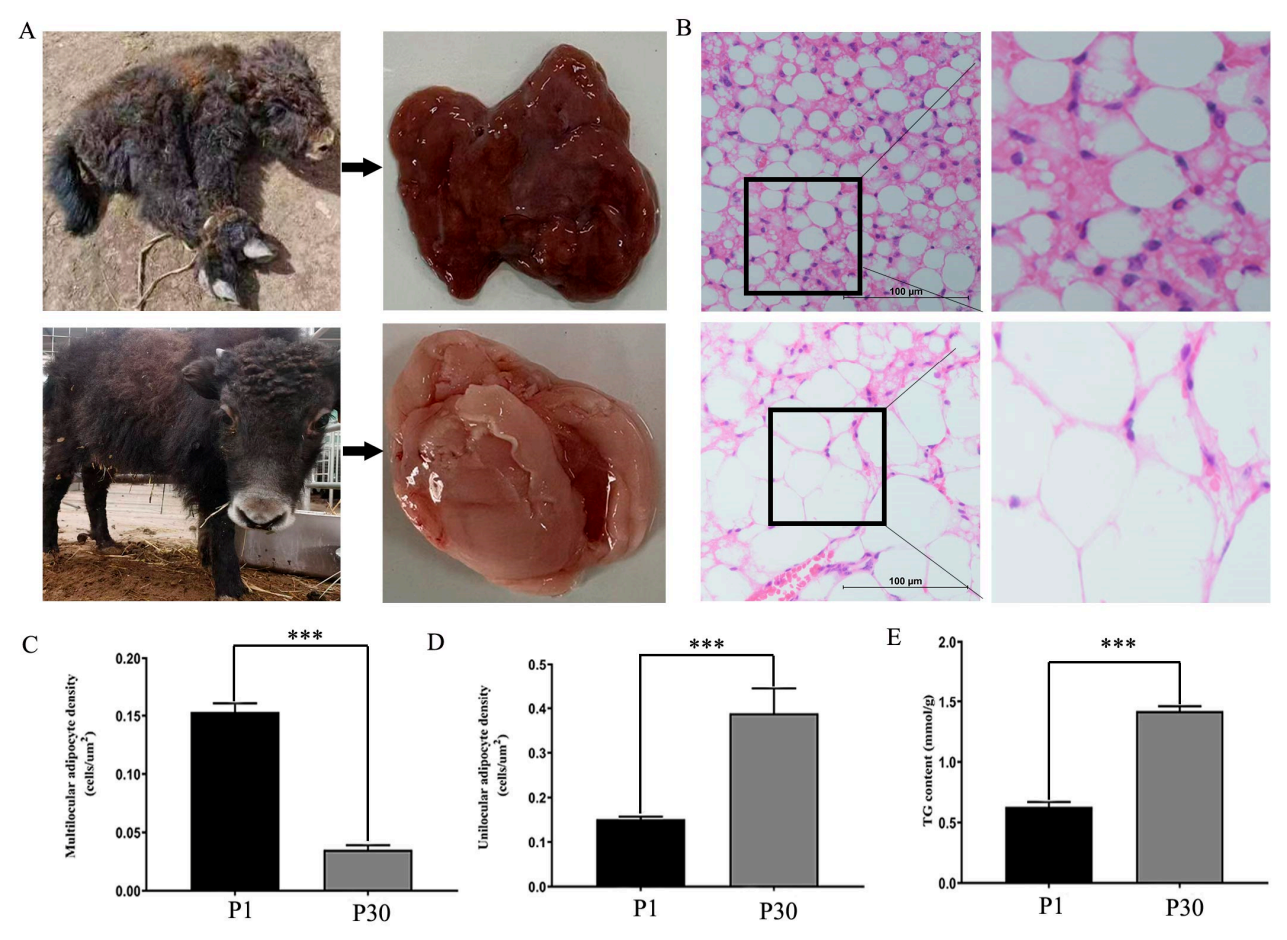
Histological morphology and lipid deposition in perirenal adipose tissue at 1-day-old (P1) and 30-day-old (P30) yak calves. (**A**) Gross morphology of perirenal adipose tissue. (**B**) H&E staining of perirenal adipose tissue. Scale bars: 100 μm. (**C**) Multilocular adipocyte density. (**D**) Unilocular adipocyte density. (**E**) Triglyceride content. Data are shown as mean ± standard error of the mean (SEM), *n* = 4 animals per group. *** indicates a significant difference at *p* < 0.05 versus the P1 group.

Gross examination revealed that the perirenal adipose tissue of yak calves changed from a reddish-brown appearance at P1 to a light pink by P30 (Figure 1A). Histological evaluation by H&E staining showed that P1 perirenal adipose tissue was predominantly composed of multilocular adipocytes. At P30, however, the majority of these multilocular adipocytes had converted into unilocular, white-like adipocytes, although a subset of multilocular adipocytes persisted (Figure 1B). Quantitative analysis confirmed a significant reduction in the density of multilocular adipocytes at P30 compared with P1 (Figure 1C, *p* < 0.05), accompanied by a marked increase in the density of unilocular adipocytes (Figure 1D, *p* < 0.05). Moreover, triglyceride (TG) content was significantly elevated in P30 adipose tissue relative to P1 (Figure 1E, *p* < 0.05). Collectively, these results indicate a phenotypic transition of perirenal adipose tissue from a brown to a white-like state during the first postnatal month, accompanied by increased lipid storage.

### 3.2. Transcriptomic Profiling Reveals Distinct Gene Expression Signatures Between P1 and P30 Adipose Tissue

Following stringent quality control of the raw sequencing data, a total of 40.44 Gb of clean bases were obtained. The average Q20 and Q30 values were 97.04% and 91.96%, respectively, and the average GC content ranged from 46.52% to 50.86% across samples (Table S2). Alignment of the clean reads to the reference genome revealed that the mapping rate for each sample exceeded 91.99% (Table S3), satisfying the requirements for subsequent analyses.

**Figure 2:**
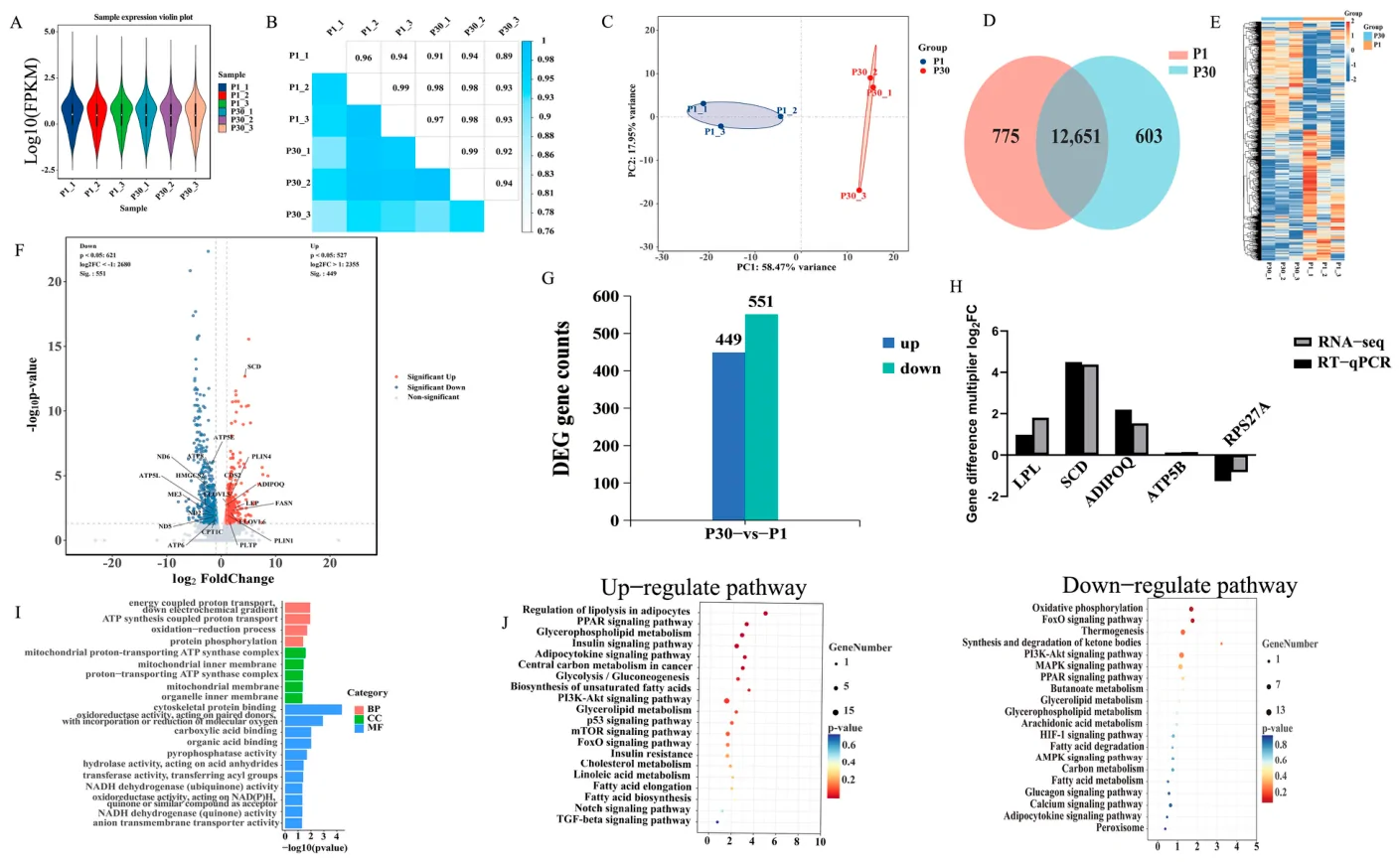
Overview of transcriptomic profiles of perirenal adipose tissue from P1 and P30 yak calves. (**A**) Violin plot of gene expression distributions. (**B**) Samples correlation coefficient plot. (**C**) PCA score plot showing the overall distribution between the two groups. (**D**) Venn diagram showing overlapping and uniquely expressed genes. (**E**) Heatmap of differentially expressed genes (DEGs). (**F**) Volcano plot of DEGs; red and blue dots represent upregulated and downregulated genes, respectively. (**G**) Bar chart showing the numbers of upregulated and downregulated DEGs. (**H**) RT-qPCR validation of five selected DEGs. Data are presented as mean ± SEM (*n* = 3 per group). *p* < 0.05 vs. P1 group. (**I**) Gene Ontology (GO) enrichment analysis of DEGs. (**J**) Kyoto Encyclopedia of Genes and Genomes (KEGG)pathway enrichment analysis of DEGs between the two groups.

The FPKM values for all genes were obtained after correcting for sequencing depth and gene/transcript length (Figure 2A). Biological reproducibility was assessed through correlation and principal component analyses. High pairwise Pearson correlation coefficients were observed among biological replicates, with intra-group correlations ranging from 0.94 to 0.99 in the P1 group and from 0.92 to 0.99 in the P30 group (Figure 2B). PCA revealed a clear separation between the P1 and P30 groups along the first principal component (PC1, 58.47% variance explained) (Figure 2C), indicating a substantial transcriptomic shift during the BAT-to-WAT transition. Venn diagram analysis identified 12,651 genes commonly expressed in both groups, alongside 775 genes uniquely expressed at P1 and 603 genes uniquely expressed at P30 (Figure 2D). Hierarchical clustering of all DEGs clearly segregated the two age groups, with the three biological replicates within each group clustering together (Figure 2E), further confirming that the sequencing data can be used for differential expression analysis.

Transcriptomic differential expression analysis identified a total of 1000 DEGs between P1 and P30, of which 551 were upregulated and 449 were downregulated (Figure 2F,G). RT-qPCR was used to validate five randomly selected DEGs (Figure 2H), comprising four upregulated and one downregulated genes. The expression patterns detected by RT-qPCR were consistent with the transcriptome sequencing results, confirming the high reliability of the DEGs expression profiles identified in this study.

To further elucidate the biological functions of DEGs, GO and KEGG enrichment analyses were performed on the DEGs identified between the two groups. GO functional classification results showed that the DEGs were primarily enriched in biological processes such as energy-coupled proton transport, ATP synthesis-coupled proton transport, and biological regulation. In cellular component categories, most DEGs were associated with the mitochondrial proton-transporting ATP synthase complex and the mitochondrial inner membrane. In molecular function categories, most DEGs were associated with NADH dehydrogenase activity (Figure 2I). KEGG enrichment analysis indicated that the PPAR pathway, glycerophospholipid metabolism pathway, insulin pathway, and adipocytokine signaling pathway were significantly enriched among the differentially expressed genes at P30, whereas thermogenesis and oxidative phosphorylation were enriched among the downregulated genes, indicating a switch from fatty acid oxidation for thermogenesis toward lipid synthesis and storage (Figure 2J). 13 thermogenesis-related genes were identified, including *ATP*5*E*, *ATP*5*L*, *ATP*6, *ATP*8, *ND*2, *ND*5, *ND*6, *HMGCS*2, *ME*3, *CPT*1*C*, *TGFB*3, *GADD*45*B* and *MAPK*13 (Table S4), and 24 lipid metabolism-related genes were identified, including *SCD*, *PLIN*1, *PLIN*4, *ADIPOQ*, *RXRA*, *PLTP*, *ELOVL*5, *ELOVL*6, *GPAM*, *CDS2*, *AGPAT*2, *ETNK*1, *DGKH*, *PHOSPHO*1, *GPD2*, *FASN*, *ADORA*1, *PRKACA*, *IRS*1, *PIK3R*1, *AKT*2, *PTGER*3, *PDPK*1 and *INSR* (Table S5). These results systematically reveal transcriptomic differences between P1 and P30 yak calves in perirenal BAT and WAT and provide insight into their potential biological functions.

### 3.3. Metabolomic Remodeling Accompanies the BAT-to-WAT Transition

**Figure 3:**
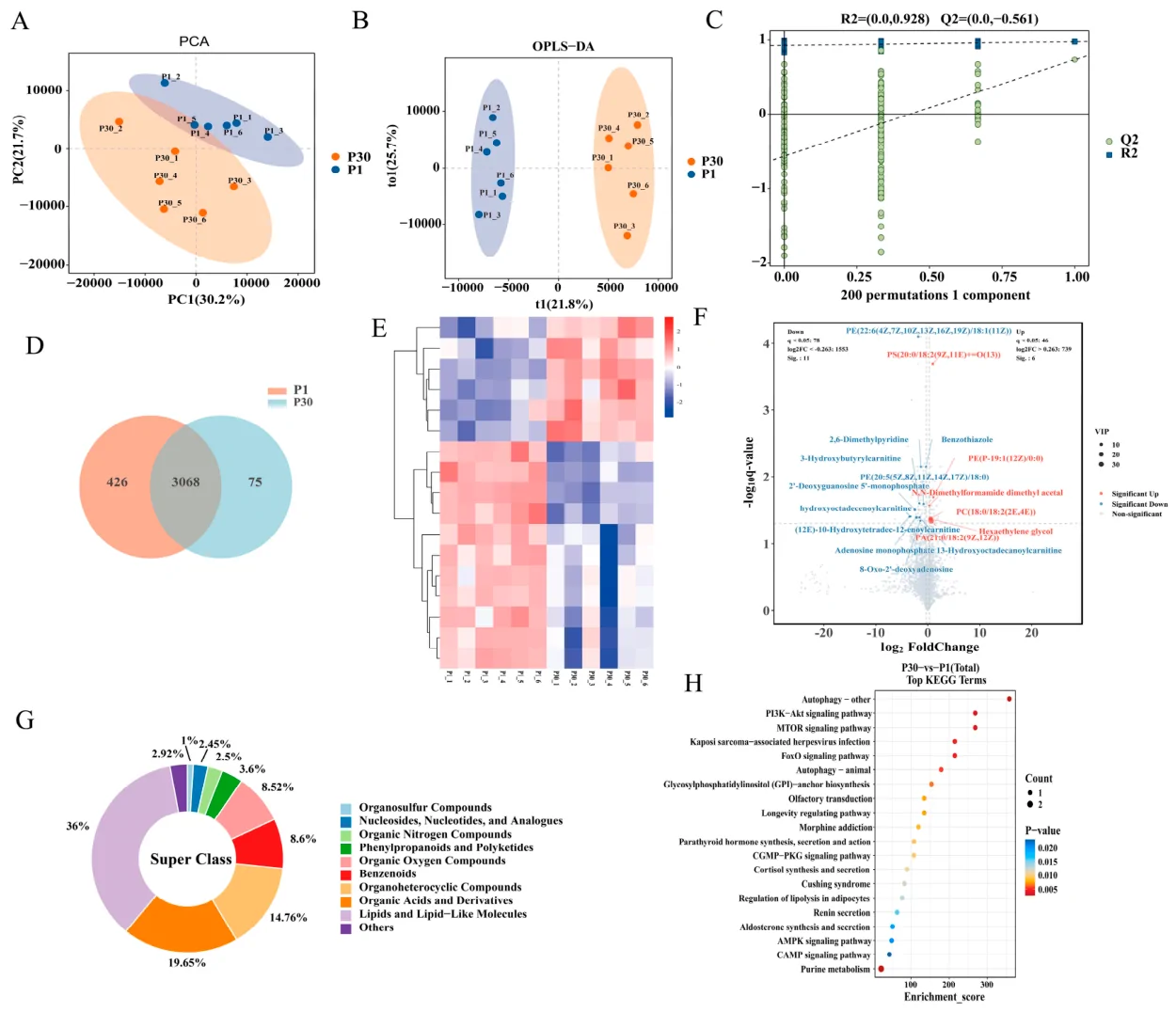
Multivariate statistical analysis of metabolomic data for perirenal adipose tissue at P1 and P30 yak calves. (**A**) PCA score plot showing the overall distribution between the two groups. (**B**) OPLS-DA score plot between the two groups. (**C**) Permutation test plot of the OPLS-DA model (200 permutations). (**D**) Venn diagram of differential metabolites (DMs). (**E**) Heatmap of DMs between the two groups. (**F**) Volcano plot of DMs; each point represents a metabolite, with red points indicating significantly upregulated metabolites and blue points indicating significantly downregulated metabolites (FC > 1.2 or FC < 1/1.2, VIP > 1.0 and q-value < 0.05). (**G**) Classification of DMs between the two groups. (**H**) KEGG enrichment analysis of DMs.

To characterize metabolic alterations during adipose tissue whitening, untargeted metabolomics was performed on perirenal adipose tissues from P1 and P30 animals. PCA showed clear separation between the two groups, with PC1 and PC2 explaining 30.2% and 21.7% of the total variation, respectively, and good intra-group clustering (Figure 3A).

Furthermore, OPLS-DA score plots indicated a clear separation between the two groups (Figure 3B), and the validity of the OPLS-DA models was assessed by 200 permutation tests. The permutation test yielded a Q-intercept of −0.561, which indicates that the model showed no overfitting (Figure 3C). The cumulative performance parameters of the original OPLS-DA model were R^2^Y = 0.978 and Q^2^ = 0.739 (Figure 3C). Differential analysis identified a total of 3068 common DMs across both groups (Figure 3D). To further determine the relationships between samples from the two groups, all DMs were subjected to clustering analysis using a heatmap. The samples from the two groups were divided into two distinct clusters, and the six biological replicates within each stage clustered together (Figure 3E). Using thresholds of FC > 1.2 or FC < 1/1.2, VIP > 1.0 and q-value < 0.05, we identified 17 DMs between the two groups, including 6 upregulated and 11 downregulated DMs at P30 (Figure 3F). Metabolite classification analysis revealed that these DMs were mainly lipids and lipid-like molecules, organic acids and derivatives, and organoheterocyclic compounds (Figure 3G).

Subsequently, KEGG pathway enrichment analysis of DMs revealed significant enrichment of pathways related to PI3K-AKT signaling, mTOR signaling, FoxO signaling, AMPK signaling, cAMP signaling, and regulation of lipolysis in adipocytes among the differential metabolites at P30 (Figure 3H). From these pathways, we annotated 6 thermogenesis-related DMs and 4 lipid metabolism-related DMs (Tables S6 and S7).

### 3.4. Integrative Analysis of the Transcriptome and Metabolome

**Figure 4:**
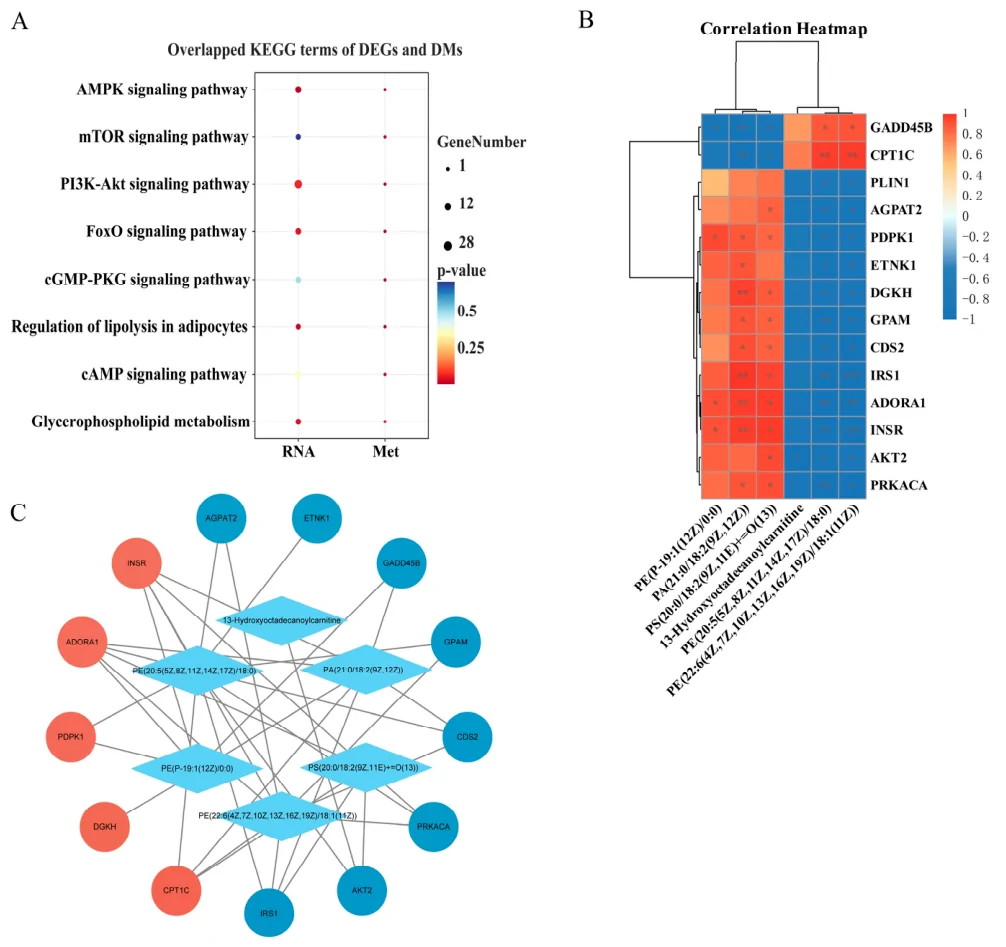
Integrated transcriptome-metabolome correlation analysis of yak perirenal adipose tissue at P1 and P30. (**A**) Integrated analysis of transcriptomic and metabolomic data. (**B**) Correlation coefficient plot of DEGs and DMs. * indicates q < 0.05, ** indicates q < 0.01, *** indicates q < 0.001. (**C**) Correlation network diagram of DEGs and DMs. Pearson correlation analysis was performed to identify gene-metabolite associations.

To investigate the association between DEGs and DMs between the two groups, we integrated the results of pathway enrichment analyses. Four pathways related to thermogenesis and lipid metabolism were commonly enriched in both omics datasets (Figure 4A), including the AMPK signaling pathway, FoxO signaling pathway, regulation of lipolysis in adipocytes, and glycerophospholipid metabolism (*p* < 0.05). A Pearson correlation analysis was performed to explore the relationships between DEGs and DMs, resulting in a total of 84 gene–metabolite pairs. To control for false-positive findings, multiple testing correction was conducted using the Benjamini–Hochberg method, and significance was determined based on the FDR-adjusted q-value. Gene–metabolite pairs with an absolute Pearson correlation coefficient (|r|) > 0.9 and an FDR-adjusted q-value < 0.05 were considered significantly correlated. Hierarchical clustering analysis further revealed consistent variation patterns of these metabolites and genes between the two groups, and a total of 14 genes and 6 metabolites were identified (Figure 4B).

Notably, among these genes, several lipid-metabolism-related genes, including *GPAM*, *AGPAT*2, *CDS*2, and *PLIN*1, showed significant positive correlations with phosphatidic acid (PA) and oxidized phosphatidylserine (PS), whereas they exhibited significant negative correlations with PE, suggesting their potential role in lipid deposition and phospholipid remodeling. In contrast, *CPT*1*C* and *GADD*45*B* exhibited significant negative correlations with PA and PS, but positive correlations with PE, implying their potential functional links to fatty-acid utilization and thermogenic metabolism (Figure 4B). Subsequently, an interaction network was constructed based on a correlation coefficient threshold of >0.9 after FDR correction (Figure 4C). Within this network, PA, PS, and PE exhibited high connectivity and formed close interactions with DEGs such as *GPAM*, *AGPAT*2, *PLIN*1, *CPT*1*C*, and *GADD*45*B*, suggesting that these genes may serve as a potential link between lipid deposition and thermogenic metabolism.

### 3.5. Fatty Acid Composition Differs Between BAT and WAT

**Figure 5:**
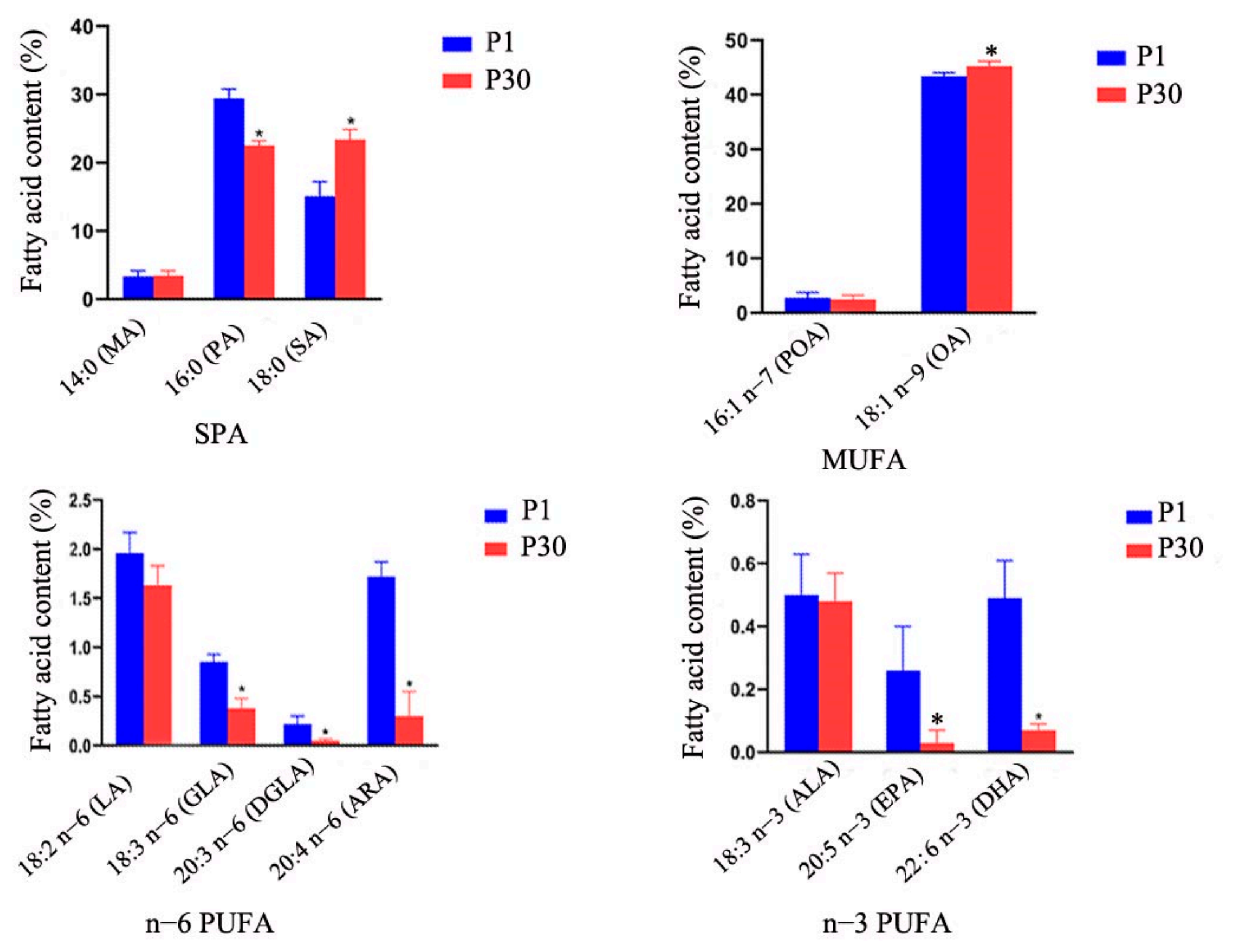
Fatty-acid compositional profiling of yak perirenal adipose tissue at P1 and P30. MA: myristic acid (14:0); PA: palmitic acid (16:0); SA: stearic acid (18:0); POA: palmitoleic acid (16:1 n-7); OA: oleic acid (18:1 n-9); ODA: octadecadienoic acid (18:2 n-3); ALA: α-linolenic acid (18:3 n-3); LA: linoleic acid (18:2 n-6); GLA: γ-linolenic acid (18:3 n-6); ARA: arachidonic acid (20:4 n-6); DGLA: dihomo-γ-linolenic acid (20:3 n-6); EPA: eicosapentaenoic acid (20:5 n-3); DHA: docosahexaenoic acid (22:6 n-3). SFA: saturated fatty acids; MUFA: monounsaturated fatty acids; PUFA: polyunsaturated fatty acids. Values are presented as mean ± SEM (*n* = 6). * *p* < 0.05.

Our RNA-seq and metabolomic analyses revealed a significant enrichment of fatty acid desaturation and degradation pathways. Analysis of the individual fatty-acyl-chain composition in the two types of adipose tissues revealed that palmitic acid (PA, 16:0), γ-linolenic acid (GLA, 18:3 n-6), dihomo-γ-linolenic acid (DGLA, 20:3 n-6), arachidonic acid (ARA, 20:4 n-6), eicosapentaenoic acid (EPA, 20:5 n-3), and docosahexaenoic acid (DHA, 22:6 n-3) were enriched in BAT, whereas stearic acid (SA, 18:0) and oleic acid (OA, 18:1 n-9) were more abundant in WAT (Figure 5, Table 1).

## 4. Discussion

BAT provides newborn mammals with a critical capacity for non-shivering thermogenesis and enables their adaptation to the cold of the extrauterine environment. In the present study, abundant multilocular adipocytes were found in the perirenal adipose tissue of P1 yak calves. In contrast, many unilocular adipocytes were found in the perirenal adipose tissue at P30. These structural changes were accompanied by a decrease in the number of multilocular adipocytes per unit area and an increase in the number of unilocular adipocytes in perirenal adipose tissue from P1 to P30. These results indicate an age-related change in adipocyte morphology within the perirenal depot, consistent with patterns reported after birth in lambs, goats and calves [10,11,12,13,16,17,18]. Interestingly, the perirenal adipose tissue of yak calves still contained typical multilocular adipocytes at P30. This contrasts with our previous study, which found that interscapular adipose tissue and subcutaneous adipose tissue from the inguinal region of yak calves were predominantly composed of white adipocytes. This suggests that the age-related shift from multilocular to unilocular adipocytes occurs at different rates among adipose depots during postnatal development in yak calves. In addition, TG content increased significantly from P1 to P30, indicating enhanced lipid storage in the perirenal depot with age. Based on these findings, we hypothesize that the persistence of multilocular adipocytes in the perirenal adipose tissue of yak calves may be associated with adaptation to the cold extrauterine environment, although thermogenic function was not directly measured in the present study. The elevation in TG content may reflect increased lipid storage during postnatal maturation, but this remains speculative.

### 4.1. Transcriptional Signatures of BAT Thermogenic Capacity

The 13 thermogenesis-related genes upregulated in BAT predominantly converge on mitochondrial oxidative phosphorylation and thermogenic capacity. BAT thermogenesis relies on fatty acid oxidation to fuel the TCA cycle and sustain uncoupled respiration, which drives electron transfer and ATP synthesis [33]. Among our candidates, *ATP*8, *ATP*5*L*, *ATP*6, and *ATP*5*E* are critical for the assembly and function of mitochondrial ATP synthase (complex V) [34,35]; their high expression in BAT likely reflects the elevated ATP turnover required for NST, consistent with reports that *ATP*6 downregulation impairs oxidative phosphorylation [36]. Similarly, *ND*2, *ND*5, and *ND*6 encode mitochondrial subunits of complex I, which catalyzes NADH oxidation and proton pumping [37]. Beyond core respiratory components, *HMGCS*2, a ketogenic enzyme strongly induced during hibernation [38], and *ME*3, which links the TCA cycle to lipid synthesis and redox balance, may support metabolic flexibility, although their precise roles in BAT remain to be elucidated. *CPT*1*C*, although known to regulate hypothalamic BAT activation via its neuronal isoform [39,40], has an undefined function within brown adipocytes themselves. Meanwhile, *TGFB*3, as a member of the transforming growth factor beta (TGF-β) gene family, plays a role in cold acclimation adaptation; cold acclimation may enhance the adaptability of *T. belangeri* to cold environments by modulating the expression of TGF-β family genes [41]. Growth arrest and DNA damage-inducible protein 45 beta (*GADD*45*B*) acts as an adaptive regulator of adipocyte senescence and systemic metabolic homeostasis that sustains adipose tissue plasticity and links DNA damage responses to adipocyte senescence and metabolic homeostasis in obesity [42]. The p38 MAPK pathway plays a key role in the activation of BAT thermogenesis [43]. Mitogen-activated protein kinase 13 (MAPK13), as a MAPK family member, may play a role in yak BAT thermogenesis. Collectively, these candidates may be involved in BAT differentiation, mitochondrial respiration, and thermogenesis in yak calves, but their specific regulatory networks require functional validation.

### 4.2. Transcriptional Signatures of WAT Lipid Storage Capacity

In contrast to the thermogenic genes upregulated in BAT, we observed significant upregulation of 24 lipid metabolism-related genes in WAT. These genes were mainly involved in fatty acid synthesis and elongation, triglyceride synthesis, lipid droplet regulation, phospholipid metabolism, and adipocyte metabolic signaling. Notably, the upregulation of *FASN*, *SCD*, *ELOVL*5, *ELOVL*6, *GPAM*, *AGPAT*2, *DGKH*, *GPD*2, *PLIN*1, *PLIN*4, *ADIPOQ*, *CDS*2, *ETNK*1, and *PHOSPHO*1 indicates coordinated changes in fatty acid synthesis, TG assembly, lipid droplet regulation, adipokine signaling, and phospholipid metabolism [44,45,46,47,48,49,50,51,52,53,54,55,56]. This coordinated transcriptional pattern is consistent with the established role of WAT as an energy storage depot and with the metabolic remodeling accompanying adipocyte maturation [44]. In particular, the increased expression of genes involved in TG synthesis and lipid droplet regulation is consistent with the enhanced TG deposition observed during the postnatal development of yak perirenal adipose tissue, supporting a transcriptional shift from thermogenic metabolism toward lipid storage during the age-related change from a brown-like to a white-like phenotype between P1 and P30.

In addition, several upregulated genes were involved in insulin- and cAMP/GPCR-related signaling pathways, including *INSR*, *IRS*1, *PIK*3*R*1, *PDPK*1, *AKT*2, *ADORA*1, *PTGER*3, and *PRKACA* [57,58,59,60,61,62,63]. These signaling pathways have previously been implicated in the regulation of glucose utilization, lipid synthesis, adipocyte survival, and lipolysis, suggesting that their coordinated upregulation may contribute to the functional maturation and lipid-storage capacity of WAT during postnatal development. The upregulation of RXRA and PLTP further supports the involvement of transcriptional regulation and lipid transport in this process [64,65]. Overall, our findings are consistent with previous studies showing that adipose tissue whitening is accompanied by enhanced lipid storage and remodeling of metabolic signaling pathways [44,57,58,59,60,61,62,63,64,65], and extend these observations to the perirenal adipose tissue of yak calves. Together, these results suggest that the age-related change from a brown-like to a white-like phenotype between P1 and P30 is characterized not only by attenuation of thermogenic characteristics but also by coordinated activation of lipid-storage and metabolic regulatory programs.

### 4.3. Integrated Analysis Identifies AMPK and FoxO Signaling as Pathways Associated with the BAT-to-WAT Transition

KEGG pathway enrichment analysis of both DEGs and DMs revealed that the AMPK and FoxO signaling pathways were commonly enriched, suggesting that these pathways may be associated with the age-related change from a brown-like to a white-like phenotype between P1 and P30. The AMPK signaling pathway has been reported to be involved in regulating energy homeostasis and adipose tissue thermogenesis [21]; the enrichment of DEGs and DMs in this pathway at P30 suggests that AMPK-related metabolic processes may be associated with this age-related phenotypic change. Meanwhile, FoxO transcription factors, which integrate insulin and growth factor signaling, have been implicated in adipocyte differentiation and metabolic reprogramming [66]. In addition to these two signaling pathways, the regulation of lipolysis in adipocytes and glycerophospholipid metabolism was also commonly enriched in both omicsdatasets. Glycerophospholipids serve as structural and functional components of cellular membranes, and their remodeling has been associated with adipocyte differentiation [56]. Collectively, these findings suggest that AMPK, FoxO, and glycerophospholipid metabolism may be involved in the age-related change from a brown-like to a white-like phenotype between P1 and P30 in yak calves.

To further characterize the molecular players underlying this transition, we performed hierarchical clustering analysis, which revealed consistent variation patterns between metabolites and genes across the two groups, and we identified 14 genes and 6 metabolites. Among these genes, several involved in lipid metabolism, including *GPAM*, *AGPAT*2, *CDS*2, and *PLIN*1, showed significant positive correlations with DMs, such as phosphatidic acid (PA) and oxidized phosphatidylserine (PS), but negative correlations with phosphatidylethanolamine (PE), suggesting their potential role in promoting lipid deposition and phospholipid remodeling. In contrast, *CPT*1*C*, a gene involved in mitochondrial fatty acid oxidation [67], and *GADD*45*B* exhibited significant negative correlations with PA and PS but positive correlations with PE, suggesting their potential associations with fatty acid utilization and thermogenesis-related metabolism. These distinct correlation patterns imply that, during the transition from P1 to P30, the identified DEGs may not only influence triglyceride (TG) storage but also participate in reshaping the fatty acid composition of membrane phospholipids, a process integral to the metabolic reprogramming of brown adipose tissue.

### 4.4. Fatty Acid Composition Reflects Functional Divergence Between BAT and WAT

BAT and iWAT alter the acyl chain composition of TG in response to cold challenge, a process often accompanied by elongation of fatty acyl chains [68]. Fatty acids serve as an important fuel for BAT NST [69]. Supplementation with omega-3 polyunsaturated fatty acids (n-3 PUFAs) in mice enhances brown adipocyte recruitment under cold-stimulated conditions [70]. Moreover, substantial evidence suggests that certain PUFAs, including eicosapentaenoic acid (EPA, 20:5 n-3) [71], docosahexaenoic acid (DHA, 22:6 n-3) [70], and linoleic acid (LA, 18:2 n-6) [72,73,74], contribute to promoting the thermogenic capacity of WAT and increasing UCP1 expression levels. Cold exposure has also been reported to significantly induce fatty acid elongation and desaturation in mouse inguinal white adipose tissue (iWAT) [75]. Interestingly, in the present study, palmitic acid (PA, 16:0), a saturated fatty acid, accounted for a relatively high proportion in BAT. PA has been shown to induce UCP1-independent mitochondrial depolarization specifically in brown adipose tissue mitochondria, suggesting a potential contribution to BAT mitochondrial function and thermogenesis [76]. In addition, several PUFAs, including γ-linolenic acid (GLA, 18:3 n-6), dihomo-γ-linolenic acid (DGLA, 20:3 n-6), arachidonic acid (ARA, 20:4 n-6), eicosapentaenoic acid (EPA, 20:5 n-3), and docosahexaenoic acid (DHA, 22:6 n-3) accounted for relatively high proportions in BAT, with the proportions of n-6 PUFA and total PUFA also being significantly higher than those observed in WAT. These coordinated changes suggest that the transition from BAT to WAT is associated with remodeling of the PUFA composition. While our data only reflect changes in fatty-acid composition, several of these PUFA species have been linked to adipose thermoregulation in other animal studies. Dietary GLA has been reported to increase UCP1 mRNA levels in brown adipose tissue and attenuate body fat accumulation [77]. In addition, EPA promotes brown and beige adipocyte recruitment, enhances thermogenic capacity, and increases UCP1 expression, while DHA has been reported to promote brown adipocyte recruitment and thermogenesis [70,71,72]. These distinct fatty acid profiles may be related to differences in mitochondrial function reflected in our transcriptomic dataset. From a biological perspective, fatty acids can serve not only as substrates for mitochondrial oxidation but also as regulators of mitochondrial activity in thermogenic adipocytes. Consistent with this possibility, our transcriptomic data revealed higher expression of genes associated with mitochondrial energy metabolism in BAT, such as *ATP*5*E* and *ND*5. *ATP*5*E* encodes a subunit of mitochondrial ATP synthase [78], whereas *ND*5 is a component of respiratory complex I, suggesting a greater potential capacity for mitochondrial oxidative phosphorylation in BAT [79]. In thermogenic adipocytes, increased electron transport can contribute to the generation of a mitochondrial proton gradient, which can subsequently be dissipated through uncoupling to produce heat rather than being used exclusively for ATP synthesis [80]. Thus, the PUFA-enriched fatty acid profile together with higher expression of mitochondrial energy metabolism-related genes may be consistent with the greater mitochondrial metabolic capacity characteristic of BAT and its thermogenic phenotype. Nevertheless, direct measurements of mitochondrial respiration, uncoupling activity, and thermogenesis were not performed in the present study; therefore, this interpretation remains speculative.

In contrast, stearic acid (SA, 18:0) and oleic acid (OA, 18:1 n-9) accounted for relatively higher proportions in WAT, suggesting that this fatty acid profile may be associated with lipid storage in this tissue. Stearic acid (SA, 18:0) supplementation has been shown to promote TG synthesis in newborn swine enterocytes [81] and lipid accumulation in bovine adipocytes [82]. A stearic acid-rich high-fat diet increased hepatic lipid accumulation in feline liver, accompanied by elevated hepatic and serum oleic acid (18:1 n-9); the higher oleic acid-to-stearic acid ratio may be related to increased SCD-1 activity, suggesting that such a diet evokes hepatic lipogenesis [83]. In our study, the relatively higher proportions of these saturated fatty acids (SFAs) and monounsaturated fatty acids (MUFAs) in WAT may be regulated by candidate lipid metabolism-related genes identified in our transcriptomic analysis, such as *SCD* and *ELOVL*5. *SCD* catalyzes the desaturation of stearic acid (SA, 18:0) to oleic acid (OA, 18:1 n-9), and its upregulation in WAT may be related to the relatively higher proportion of oleic acid (OA, 18:1 n-9), which is esterified into triglycerides for storage. The distinct fatty acid profiles between BAT and WAT suggest a functional dichotomy: BAT contains relatively higher proportions of PUFAs that have been reported to support mitochondrial respiration and thermogenic signalling, whereas WAT contains relatively higher proportions of SFAs and MUFAs suited for TG storage.

It should be noted that changes in fatty acid composition may also be influenced by postnatal maturation and diet, in addition to adipose tissue functional remodelling. Therefore, the observed differences between BAT and WAT cannot be attributed solely to tissue-specific metabolic remodelling, and the potential contributions of dietary fatty acid supply and developmental maturation should be considered when interpreting these results.

### 4.5. Limitations of This Study

Several limitations of the present study should be acknowledged. First, the relatively small sample size may limit the statistical power and generalizability of the findings, and the selected postnatal time points may not fully capture the continuous and dynamic process of BAT-to-WAT transition in yak calves. Second, the fatty acid data primarily reflect relative proportions rather than absolute concentrations or fluxes; therefore these results should be interpreted as differences in lipid composition rather than as direct evidence of altered fatty acid metabolism. Third, although transcriptomic and metabolomic analyses identified pathways and molecular features associated with adipose tissue remodeling, direct measurements of thermogenic activity and mitochondrial function were not performed. Accordingly, the functional implications of these molecular changes should be interpreted with caution. In addition, postnatal maturation and diet may also contribute to the observed changes in adipose tissue composition and metabolism and the effects of postnatal muturation and diet could not be completely separated from those of developmental in the present study. Future studies with larger sample sizes, additional developmental time points, and direct measurements of thermogenesis and mitochondrial function will be valuable for further elucidating the mechanisms underlying BAT-to-WAT transition in yak calves.

## 5. Conclusions

**Figure 6:**
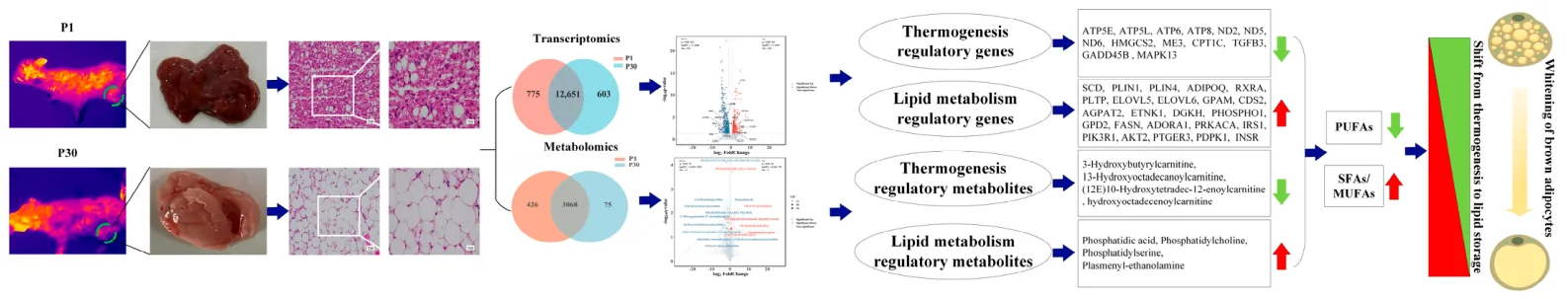
Schematic summary of the age-related change from a brown-like to a white-like phenotype in perirenal adipose tissue of yak calves. From P1 to P30, perirenal adipose tissue undergoes a phenotypic transition from multilocular to unilocular adipocytes, accompanied by changes in gene expression and metabolite profiles. BAT is characterized by high expression of thermogenesis-related genes (e.g., *ATP*5*E*, *ATP*5*L*, *ATP*6, *ATP*8, *ND*2, *ND*5, *ND*6, *HMGCS*2, *ME*3, *CPT*1*C*, *TGFB*3, *GADD*45*B*, and *MAPK*13) and enrichment of PUFAs (EPA, DHA), supporting oxidative phosphorylation and non-shivering thermogenesis. WAT is characterized by upregulation of lipid metabolism-related genes (e.g., *SCD*, *PLIN*1, *PLIN*4, *ADIPOQ*, *RXRA*, *PLTP*, *ELOVL*5, *ELOVL*6, *GPAM*, *CDS2*, *AGPAT*2, *ETNK*1, *DGKH*, *PHOSPHO*1, *GPD2*, *FASN*, *ADORA*1, *PRKACA*, *IRS*1, *PIK3R*1, *AKT*2, *PTGER*3, *PDPK*1, and *INSR*) and enrichment of SFAs/MUFAs (stearic acid, oleic acid), favoring triglyceride storage and membrane remodeling. ↑, upregulation; ↓, downregulation.

This study characterizes the postnatal transition of perirenal adipose tissue from a brown-like to a white-like phenotype in yak calves and identifies the key molecular and metabolic changes associated with this process. Our results demonstrate that perirenal adipose tissue of yak calves at P1 is predominantly composed of multilocular adipocytes, whereas by P30 most adipocytes have become unilocular, although some multilocular adipocytes remain. Triglyceride content increased from P1 to P30. We identified coordinated changes in thermogenesis-related genes, lipid metabolism-related genes, and differential metabolites between BAT and WAT in yak calves (Figure 6). In addition, BAT showed higher relative proportions of n-3 PUFAs, including EPA (20:5 n-3) and DHA (22:6 n-3), whereas WAT showed higher relative proportions of stearic acid (SA, 18:0) and oleic acid (OA, 18:1 n-9). These results provide a basis for further study of the phenotypic changes in yaks calves from brown-like to white-like adipose tissue. However, owing to the relatively small sample size and the limitation of comparing only two postnatal developmental stages, the present work cannot confirm causal regulatory relationships or direct physiological functions. Further research is needed to clarify how the identified genes and metabolites regulate the transition from multilocular to unilocular adipocytes after birth, as well as the molecular mechanisms underlying this cellular transition; which may improve our understanding of adaptation to the cold extrauterine environment in plateau ruminants.

## Figures and Tables

**Table 1:** Fatty acid composition and content in perirenal adipose tissue of yak calves at the BAT (day 1) and WAT (day 30) stages.

Fatty Acids	Day 1	Day 30	*p*-Value
C14:0	3.35 ± 0.80	3.45 ± 0.69	0.850
C16:0	29.43 ± 1.34	22.51 ± 0.72 **	0.000
C18:0	15.08 ± 2.14 **	23.40 ± 1.49	0.001
SFA	47.85 ± 1.55	49.36 ± 0.57	0.228
C16:1n-7	2.76 ± 0.97	2.47 ± 0.77	0.661
C18:1n-9	43. 40 ± 0.65 *	45.22 ± 0.92	0.025
MUFA	46.15 ± 1.62	47.70 ± 0.56	0.236
C18:2n-6	1.96 ± 0.21	1.63 ± 0.20	0.062
C18:3n-6	0.85 ± 0.08	0.38 ± 0.10 **	0.000
C20:3n-6	0.22 ± 0.08	0.05 ± 0.02 **	0.003
C20:4n-6	1.72 ± 0.15	0.30 ± 0.25 **	0.000
n-6 PUFA	4.75 ± 0.30	2.37 ± 0.12 **	0.000
C18:3n-3	0.50 ± 0.13	0.48 ± 0.09	0.812
C20:5n-3	0.26 ± 0.14	0.03 ± 0.04	0.094
C22:6n-3	0.49 ± 0.12	0.07 ± 0.02 *	0.022
n-3 PUFA	1.25 ± 0.38	0.57 ± 0.13	0.082
PUFA	6.00 ± 0.08	2.94 ± 0.22 **	0.000
n-3/n-6 PUFA	0.27 ± 0.09	0.24 ± 0.05	0.631
Total	100.00	100.00	

Values with * indicate significant differences (*p* < 0.05), and ** indicate highly significant differences (*p* < 0.01).

## Data Availability

Raw metabolomic data were deposited in the National Genomics Data Center (NGDC, CNCB) under accession number OMIX016933 (https://ngdc.cncb.ac.cn/omix/preview/RfkfaAah, accessed on 8 October 2026). The raw transcriptomic sequencing data were deposited in the NCBI SRA database (PRJNA1359552).

## Supplementary Materials

The following supporting information can be downloaded at: https://www.mdpi.com/article/10.3390/ani16203219/s1. Table S1. Primer sequence information. Table S2. Quality analysis of transcriptome sequencing data. Table S3. Summary of read mapping statistics against the reference genome. Table S4. Summary of candidate DEGs identified from thermogenesis related pathways in perirenal adipose tissue of yak calves at the BAT (day 1) and WAT (day 30) stages. Table S5. Summary of candidate DEGs identified from lipid metabolism related pathways pathways in perirenal adipose tissue of yak calves at the BAT (day 1) and WAT (day 30) stages. Table S6. Summary of candidate differential metabolites screened from thermogenesis-related pathways in perirenal adipose tissue of yak calves at the BAT (day 1) and WAT (day 30) stages. Table S7. Summary of candidate differential metabolites identified from lipid metabolism-related pathways in perirenal adipose tissue of yak calves at the BAT (day 1) and WAT (day 30) stages.
