# Supplementary material for: Integrated Transcriptomic and Metabolomic Analyses Reveal the Whitening of Perirenal Brown Adipose Tissue in Yak Calves
Source: Animals (Basel). 2026 Oct 13;16(20):3219. doi: 10.3390/ani16203219 (PMC13700673; doi:10.3390/ani16203219)
Supplement: Supplementary file 1 [file animals-16-03219-s001.zip › animals-4540437-supplementary.pdf]

Table S1 Primer sequence information

Gene	Primer sequences（5'-3'）	Product length/bp	Annealing temperature/ ℃	GenBank accession No.
ATP5B	F: GGTGAGAGCACAGTAAGGACCATTG<br>R: GCACCAGAATCCAGCACTTTCTGAC	80	60	NM_175796.3
RPS27A	F: CAAAAGGAGTCCACTCTTCA<br>R: CGACTGATTTTGCCATTCTC	164	54	NM_174778.1
LPL	F: GAGGACACTTGCCACCTCAT<br>R: TACATTCCTGTCACCGTCCA	113	55	XM_014480300.1
ADIPOQ	F: TGTGGCTCTGATTCCACACC<br>R: CGGGGGATCTTGCATGTTGT	108	60	XM_005910767.2
SCD	F: TGGCGTTCCAGAATGACGTT<br>R: GAAGAAAAAGCCACGTCGGG	107	60	XM_005892055.2
β-actin	F: TCCTGGGTATGGAGTCCTG<br>R: TGATCTTCATCGTGCTTGG	186	60	XM_005887322.2

Table S2 Quality analysis of transcriptome sequencing data

sample	raw reads per sample	Raw bases/GB	clean reads per sample	Clean bases/GB	Error rate/%	Q20/%	Q30/ %	GC_pct /%
P1_1	46330650	6.95G	44483002	6.67G	0.03	96.96	91.64	46.52
P1_2	49990838	7.5G	48429336	7.26G	0.03	97.21	92.38	49.22
P1_3	45336784	6.8G	42210558	6.33G	0.03	96.15	90.1	47.81
P30_1	47819800	7.17G	46441926	6.97G	0.03	97.3	92.41	49.62
P30_2	45653214	6.85G	43609598	6.54G	0.03	97.33	92.67	49.75
P30_3	46583984	6.99G	44496386	6.67G	0.03	97.27	92.54	50.86

Table S3 Summary of read mapping statistics against the reference genome

sample	total_reads	total_map	unique_map	proper_map
P1_1	44483002	41745946(93.85%)	40216495(90.41%)	37435978(84.16%)
P1_2	48429336	45241585(93.42%)	43910101(90.67)	40649134(83.93)
P1_3	42210558	38829972(91.99%)	37587594(89.05%)	33393952(79.11%)
P30_1	46441926	43027290(92.65%)	41854584(90.12%)	39081642(84.15%)
P30_2	43609598	40286017(92.38%)	39204302(89.9%)	36215208(83.04%)
P30_3	44496386	40973023(92.08%)	39863545(89.59%)	36998782(83.15%)

Table S4 Summary of candidate DEGs identified from thermogenesis-related pathways in perirenal adipose tissue of yak calves at the BAT (day 1) and WAT (day 30) stages

Source pathway	Gene ID	Gene symbol	Expression pattern	Biological function
Thermogenesis	3119744	*ATP8*	P1>P30	Mediates mitochondrial proton-leak-based thermogenesis
	3126314	*ND6*		Regulates mitochondrial complex I activity
	102271531	*ATP5L*		Modulates inner-mitochondrial-membrane permeability
	3119753	*ND2*		Facilitates mitochondrial electron-transport chain
	3126313	*ND5*		Regulates mitochondrial respiratory remodelling
	3119742	*ATP6*		Constitutes the ion channel of ATP synthase
Oxidative phosphorylation	106701624	*ATP5E*	P1>P30	Participates in mitochondrial ATP-synthase activity
PPAR signaling pathway	102273324	*HMGCS2*	P1>P30	Catalyses the rate-limiting step of ketone-body synthesis
	102287267	*ME3*		Participates in mitochondrial malate-acid metabolism
AMPK signaling pathway	102287746	*CPT1C*	P1>P30	Catalyses fatty-acid transport into mitochondria
FoxO signaling pathway	102265567	*TGFB3*	P1>P30	Thermogenesis suppressor
	102266978	*GADD45B*		Lipogenesis inhibitor
	102271030	*MAPK13*		Promotes adipocyte lipolysis and browning

Table S5 Summary of candidate DEGs identified from lipid metabolism-related pathways pathways in perirenal adipose tissue of yak calves at the BAT (day 1) and WAT (day 30) stages

Source pathway	Gene ID	Gene symbol	Expression pattern	Biological function
PPAR signaling pathway	102280846	*SCD*	P30>P1	Catalyzes fatty-acid desaturation
	102278159	*PLIN4*		Regulates lipid-droplet formation
	102271886	*ADIPOQ*		Mediates adipocyte lipid metabolism
	102265047	*RXRA*		Participates in adipogenic transcriptional regulation
	102280310	*PLTP*		Facilitates lipid transportation inside adipose cells
Biosynthesis of unsaturated fatty acids	102272645	*ELOVL5*	P30>P1	Catalyzes long-chain fatty acid elongation
	102285624	*ELOVL6*		Promotes saturated fatty acid synthesis
Glycerophospholipid metabolism	102281470	*GPAM*	P30>P1	Triacylglycerol synthesis
	102279501	*CDS2*		Phospholipid synthesis
	102277646	*AGPAT2*		Glycerolipid biosynthesis
	102269833	*ETNK1*		Catalyzes ethanolamine lipid metabolism
	102268254	*DGKH*		DAG-PA conversion
	102281872	*PHOSPHO1*		Regulates lipid phosphate conversion
	102277350	*GPD2*		Participates in glycerolipid formation
Fatty acid biosynthesis	102278267	*FASN*	P30>P1	Catalyzes de novo fatty acid synthesis

Regulation of lipolysis in adipocytes	102268681	*ADORA1*	P30>P1	Inhibits lipolysis, promotes lipid accumulation
	102281757	*PRKACA*		Triggers lipolysis via phosphorylating PLIN1
	102284664	*IRS1*		Transduces insulin-PI3K-AKT signals
	102266922	*PIK3R1*		Activates PI3K-AKT pathway for lipid synthesis
	102275199	*AKT2*		Promotes lipid deposition and represses lipolysis
	102285336	*PLIN1*		Regulates lipolysis and lipid storage
	102277668	*PTGER3*		Suppresses lipolysis by lowering cAMP
FoxO signaling pathway	102264327	*PDPK1*	P30>P1	Phosphorylates and activates AKT
	102285317	*INSR*		Initiates insulin-dependent lipogenic signaling

Table S6 Summary of candidate differential metabolites screened from thermogenesis-related pathways in perirenal adipose tissue of yak calves at the BAT (day 1) and WAT (day 30) stages

Source pathway	ID	Metabolite symbol	Expression pattern	Biological function
	1.11_248.1492m/z	3-Hydroxybutyrylcarnitine		Ketone-related carnitine
	10.02_444.3680m/z	13-Hydroxyoctadecanoylcarnitine		Long-chain FA transport

Thermogenesis-related	8.03_386.2897m/z	(12E)-10-Hydroxytetradec-12-enoylcarnitine	P1>P30	Medium-chain FA transport
	9.67_486.3440m/z	hydroxyoctadecenoylcarnitine		Unsaturated FA transport
	10.44_834.5291m/z	Phosphatidylethanolamine(22:6(4Z,7Z,10Z,13Z,16Z,19Z)/18:1(11Z))		PUFA-enriched phospholipid
	10.42_810.5307m/z	Phosphatidylethanolamine(20:5(5Z,8Z,11Z,14Z,17Z)/18:0)		PUFA-containing phospholipid

Table S7 Summary of candidate differential metabolites identified from lipid metabolism-related pathways in perirenal adipose tissue of yak calves at the BAT (day 1) and WAT (day 30) stages

Source pathway	ID	Metabolite symbol	Expression pattern	Biological function
Lipid deposition-related	13.45_760.5836m/z	Phosphatidic acid(21:0/18:2(9Z,12Z))	P30>P1	Lipid-deposition mediator
	13.45_786.5988m/z	Phosphatidylcholine(18:0/18:2(2E,4E))		Lipid-droplet formation
	10.50_812.5432m/z	Phosphatidylserine(20:0/18:2(9Z,11E)+=O(13))		Adipogenic signaling
	11.25_478.3289m/z	Phosphatidylethanolamine(P-19:1(12Z)/0:0)		Lipid storage regulator
